# The dysregulation of mitochondrial homeostasis–related genes could be involved in the decrease of mtDNA copy number in systemic lupus erythematosus patients

**DOI:** 10.1007/s12026-024-09535-z

**Published:** 2024-09-04

**Authors:** Giada De Benedittis, Andrea Latini, Chiara Morgante, Carlo Perricone, Fulvia Ceccarelli, Giuseppe Novelli, Lucia Novelli, Cinzia Ciccacci, Paola Borgiani

**Affiliations:** 1 Department of Biomedicine and Prevention, Genetics Section, University of Rome Tor Vergata, 00133 Rome, Italy; 2 Rheumatology, Department of Medicine, University of Perugia, Piazzale Giorgio Menghini, 1, 06129 Perugia, Italy; 3 Lupus Clinic, Rheumatology, Department of Internal Medicine, Sapienza University of Rome, Rome, Italy; 4 UniCamillus, Saint Camillus International University of Health Sciences, 00131 Rome, Italy; 5 Department of Pharmacology, School of Medicine, University of Nevada, Reno, USA

**Keywords:** Systemic lupus erythematosus, MtDNA copy number, Mitochondrial homeostasis genes

## Abstract

Systemic lupus erythematosus (SLE) is a chronic multifactorial autoimmune disease. It is now widely demonstrated that oxidative stress (OS) plays an important role in the modulation of the pathogenesis of this disease. Mitochondrial DNA (mtDNA) is highly vulnerable to OS and it is known a decrease of mtDNA copy number in SLE patients. However, to date, it has not been investigated if this decrease is associated with a dysregulation of mitochondrial homeostasis genes. Our aim is to evaluate the amount of mtDNA copy number and the expression of the genes more involved in the mitochondrial homeostasis pathways, in peripheral blood mononuclear cells (PBMCs) of SLE patients and healthy controls. We analysed the amount of mtDNA in PBMCs of 72 SLE patients and 61 healthy controls by qPCR. Then, we investigated the expression variability of *TFAM* and *SIRT1* (biogenesis), *MFN1* and *MFF* (fusion/fission) and *PRKN2* (mitophagy) genes in a subgroup of SLE patients and healthy controls. Interestingly, we have observed a highly significant decrease in mtDNA copies in SLE patients compared to healthy controls (*P* < 0.0001). In addition, we have shown that the expression levels of *SIRT1*, *MFN1* and *PRKN2* genes were significantly decreased in SLE patients with respect to healthy controls (*P* = 0.00001 for *SIRT1*, *P* = 0.0150 for *MFN1* and *P* = 0.0009 for *PRKN2*). Lastly, we have reported a positive correlation between *PRKN2* expression level and mtDNA copy number (*P* = 0.019, *r* = 0.475). In conclusion, our data confirm the impairment of mtDNA copy number in the disease and show for the first time a dysregulation of the mitochondrial homeostasis genes. These results could provide additional support to the important role of mitochondria in SLE development.

## Introduction

Systemic lupus erythematosus (SLE, MIM: 152,700) is a chronic multifactorial autoimmune disease, characterized by a breakdown of immune tolerance and subsequent production of autoantibodies against nucleic self-antigens [1]. It is known that T cells play a primary role in SLE pathogenesis, amplifying and sustaining the chronic autoimmune response by several immunological mechanisms [2].

It is now widely recognized that oxidative stress (OS) is involved in the pathogenesis of several autoimmune diseases, including SLE, as it contributes to immune system dysregulation, organ damage and lethal comorbidities [3]. Different studies have in fact described increased levels of 8-hydroxy-2′-deoxyguanosine, a common oxidative DNA damage marker, in blood cells of SLE patients [4]. In addition, oxidative stress products, such as lipid peroxides and oxidized proteins, are significantly increased in serum and in lymphocytes of SLE patients [5–7]. Moreover, SLE T cells are characterized by a persistent mitochondrial hyperpolarization leading to a decrease of ATP production, an increase of reactive oxygen species (ROS) and a depletion of reduced glutathione (GSH) that contribute to an increase in the OS typical of this disease [8, 9]. The generation of ROS occurs mainly at the electron transport chain located on the mitochondrial membrane, during the oxidative phosphorylation process. Mitochondria are highly dynamic organelles, and the preservation of their number and morphology is finely regulated by a cycle of production (biogenesis), morphology (homeostasis results in impaired mitochondrial transport, which could increase the production fusion/fission) and degradation (mitophagy) [10]. The dysregulation of mitochondrial homeostasis results in impaired mitochondrial transport, which could increase the production of ROS. It has been shown that persistent productions of ROS and pro-inflammatory cytokines sustain with each other [11], resulting in tissue and organ inflammation and immune cascade induction, found in autoimmune diseases such as SLE [12]. In fact, it has been demonstrated that the imbalance in mitochondrial homeostasis can lead to the development of autoimmune rheumatic diseases, including SLE [13].

Moreover, each mitochondrion contains multiple copies of mitochondrial DNA (mtDNA), which is highly vulnerable to OS, as it can induce both qualitative and quantitative changes. A decrease in mtDNA copy number in the pathogenesis of SLE has already been reported in the literature [14, 15]. However, to date, it has not been shown if this decrease is associated with a dysregulation of mitochondrial homeostasis genes in SLE patients. After a careful analysis of the literature, we selected TFAM and SIRT1 genes as biomarkers for the biogenesis process, MFN1 and MFF for fusion/fission processes and PRKN2 for the mitophagy process. In fact, it is known that SIRT1 promotes mitochondrial biogenesis through the induction of the transcription factor PGC-1α which drives mtDNA transcription/replication [16], while TFAM is essential for the maintenance and transcription of mtDNA [17]. MFN1 is one of the central proteins that binds and promotes membrane fusion of mitochondria [18], while MFF is an integral membrane protein of the outer mitochondrial membrane, involved in the fission process and in mitochondrial fragmentation [18]; lastly, PRKN encodes an E3 ubiquitin ligase that has been described as a key regulator of mitophagy [19]. Moreover, some of these genes were also investigated in Sjögren’s syndrome patients, another autoimmune disease characterized by increased oxidative stress and impaired mitochondrial function [20].

Considering these elements, this study aimed to evaluate the amount of mtDNA copy number in peripheral blood mononuclear cells (PBMCs) of patients with SLE and healthy controls to confirm possible alterations. Afterward, in two subgroups of patients and control subjects, we aimed to investigate differences in the expression of the most common genes involved in the mitochondrial homeostasis pathway: *TFAM* and *SIRT1* (biogenesis), *MFN1* and *MFF* (fusion/fission) and *PRKN2* (mitophagy).

## Materials and methods

### Sample collection

Seventy-two adult SLE patients, diagnosed according to the 1997 revised criteria of the American College of Rheumatology [21], were enrolled from the Lupus Clinic of the Rheumatology Unit at the Sapienza University in Rome (Italy). The study protocol required a comprehensive physical examination and the gathering of clinical and laboratory data on a standardized electronic record, according to the protocol. A complete description of clinical and laboratory exams of patients was reported in a previous paper [22]. Demographic, clinical and laboratory characteristics of patients were reported as percentage or mean ± standard deviation in Table 1.

**Table 1:** Clinical and laboratory data of the SLE patients

	SLE (*n* = 72)	SLE subgroup (*n* = 30)
Age (years ± SD)	45.64 ± 11.98	47.37 ± 12.94
Age at onset (years ± SD)	31.88 ± 13.49	33.20 ± 14.11
Disease duration (years ± SD)	16.97 ± 8.40	14.50 ± 8.32
Sex (% female)	92.9%	93.3%
Musculo-skeletal involvement	45.7%	40.0%
Photosensitivity	72.5%	71.4%
Malar rash	52.9%	33.3%
Aphthous/ulcers	14.6%	14.3%
Pericarditis	20.7%	20.0%
Pleurisy	11.6%	6.7%
Nephritis	32.9%	23.3%
Anaemia	40.7%	46.7%
Leukopaenia	47.9%	30.8%
Thrombocytopaenia	22.2%	16.7%
ANA	92.5%	100%
Anti-dsDNA	56.5%	50.0%
Anti-Sm	15.4%	21.4%
Anti-RNP	16.9%	25.0%
Anti-Ro/SSA	32.3%	46.4%
Anti-La/SSB	12.3%	17.9%
Anti-CL IgG or IgM	44.8%	44.8%
Anti-β2GPI IgG or IgM	20.3%	20.0%
LAC	31.6%	33.3%
C3 (below normal values)	42.1%	31.0%
C4 (below normal values)	40.4%	37.9%

Quantitative data are expressed as mean and standard deviation (SD); dichotomous data are expressed as a percentage. *SLE*, systemic lupus erythematosus; *ANA*, antinuclear antibodies; *Anti-dsDNA*, anti-double-stranded DNA; *Anti-RNP*, ribonucleoprotein antibodies; *Anti-CL*, anticardiolipin antibodies; *Anti-β2GPI*, anti-β2 glycoprotein I; *Ig*, immunoglobulin; *LAC*, lupus anticoagulant; *C3*, complement 3; *C4*, complement 4

Sixty-one age-matched healthy subjects (median age, 59.26 years) were also enrolled as controls. Blood sample from all subjects was obtained by standard antecubital venepuncture and stored at − 20 °C until usage.

All participants provided the informed consent for the use of their clinical and laboratory data for study purposes. The study was carried out in accordance with the Declaration of Helsinki.

### Mitochondrial DNA copy number evaluation

Nuclear and mitochondrial DNA was extracted from peripheral blood mononuclear cells (PBMCs) using a Qiagen blood DNA mini kit. DNA quality and concentration were evaluated by NanoDrop ND-1000 Spectrophotometer (Euro-Clone).

mtDNA copy number analysis was performed as described by Rooney [23]. The quantification of mtDNA copy number was determined using a quantitative real-time method (qPCR) through the simultaneous amplification of both the genomic HGB gene (haemoglobin subunit beta) and the mitochondrial ND1 gene (NADH dehydrogenase, subunit 1). The reactions were performed with ABI 7500 Fast Real-time PCR System (Applied Biosystems, Foster City, CA, USA). Primers amplifying nuclear and mitochondrial DNA regions were taken from the literature [19]. Each reaction was performed in triplicate. The threshold cycle (Ct) values for the nuclear HGB gene and mitochondrial ND1 gene were concurrently determined in each sample during the same qPCR run. The difference in the average Ct number values was used for the measurement of the relative content of the mtDNA copy number in the leukocytes of each subject, according to the following equation: 2 × 2^(Ct (HGB)−Ct (ND1))^ [24].

### RNA extraction and qRT-PCR analysis

To detect the expression levels of *TFAM*, *SIRT1*, *MFN1*, *MFF* and *PRKN2* genes, 30 SLE patients and 15 healthy controls were randomly selected from the first cohort. PBMCs were isolated from blood using density gradient centrifugation with Ficoll-Paque (GE Healthcare). Then, total RNA was extracted from PBMCs using TRIzol reagent (Ambion, CA, USA) protocol and stored at − 80 ˚C until RNA extraction. The concentration and purity of isolated RNA were evaluated using a NanoDrop ND-1000 Spectrophotometer (Euroclone). Synthesis of the first-strand cDNA was conducted using the High-Capacity cDNA Reverse Transcription Kit (Applied Biosystems, Waltham, MA, USA) according to the manufacturer’s instructions.

Expression analysis was performed by RT-qPCR using ABI 7500 Fast Real-time PCR System (Applied Biosystems, Foster City, CA, USA) (SYBR Green Assay, Applied Biosystems) and specific primer pairs for each gene analysed. The primer sequences are available upon request. Each sample was analysed in triplicate. The relative difference in gene expression levels was calculated with the 2^−ΔΔCt^ method, using β-Actin as endogenous control. Data are reported as mean values ± standard deviation.

### Statistical analysis

The distribution of the expression data was firstly assessed using the Kolmogorov–Smirnov test. Accordingly, the mean mtDNA copy number and gene expression levels between different phenotypic groups were compared using the *t*-test or the Mann–Whitney *U*-test. A *p*-value of ≤ 0.05 was considered statistically significant in this study. Possible linear correlations between mtDNA copy number and selected genes were assessed by Pearson correlation analysis. Data are shown as mean ± standard deviation. Statistical analysis was conducted using SPSS program ver. 19 (IBM Corp., Armonk, NY, USA), while all graphs were performed by GraphPad Prism 9 (GraphPad Software, USA).

## Results

### Clinical characteristics of participants

A total of 72 SLE patients and 61 healthy controls were included in this study. The SLE group is mostly composed of females (92.9%) with a median age of 45.64 ± 11.98 years and an age at onset of 31.88 ± 13.49 years. Antinuclear antibodies (ANA) were detectable in most of patients (92.5%) while anti-dsDNA antibodies were detectable in 56.5% of patients. Hypocomplementemia was present in 42.1% of patients. At the time of enrolment, lupus nephritis and arthritis were the most clinical manifestations of SLE (32.9% and 45.7%, respectively).

### Mitochondrial DNA copy number

The mtDNA copy number was analysed in the PBMCs of 72 SLE patients and 61 healthy controls. PBMCs were studied because SLE is a systemic pathology, characterized by a breakdown in immune tolerance, production of autoantibodies and an inflammatory state that therefore involves several different cell populations of the immune system.

**Fig. 1:**
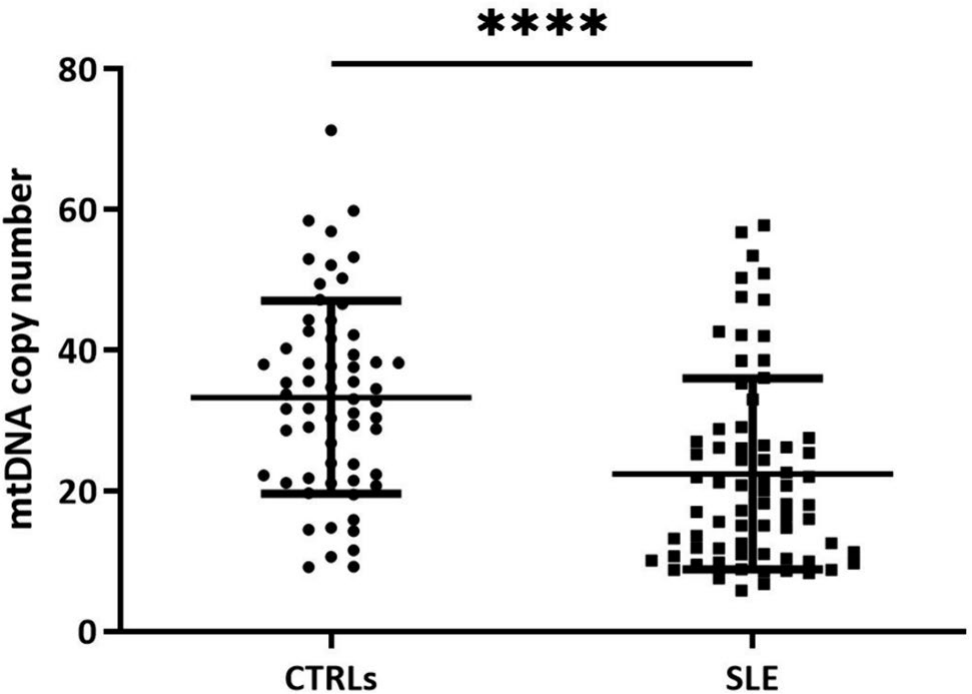
Comparison of mtDNA copy number between healthy controls (CTRLs) and patients with Systemic Lupus Erythematosus (SLE). ****P < 0.0001

As shown in Fig. 1, SLE patients exhibited a significantly lower amount of mtDNA copy number compared to the healthy controls (*P* < 0.0001) (Fig. 1).

**Fig. 2:**
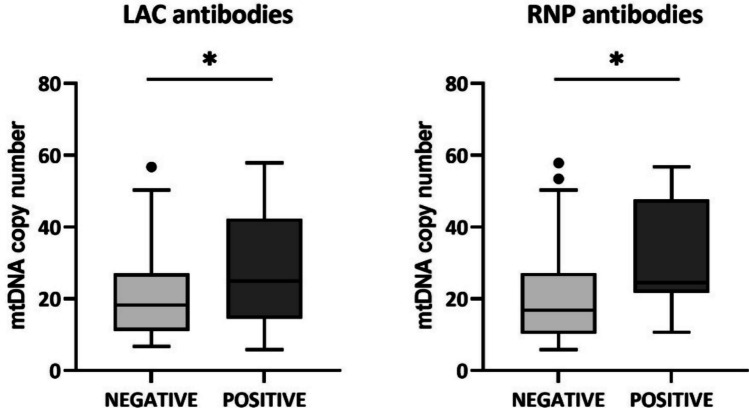
Comparison of mtDNA copy number between negative and positive patients for anti-LAC and anti-RNP antibodies. *P < 0.05

We also evaluated the possible associations of mtDNA copy number with patients’ clinical characteristics. We observed that patients positive for antiphospholipid antibodies (LAC) and antibodies to ribonucleoprotein (RNP) showed a significantly higher mean value of mtDNA copy number (*P* < 0.05) (Fig. 2).

### Expression levels of mitochondrial homeostasis genes

To better investigate changes of mitochondrial homeostasis in PBMCs of SLE patients and healthy controls, we subsequently analysed the expression levels of genes involved in mitochondrial biogenesis (*TFAM*, *SIRT1*), fusion and fission pathway (*MFN1* and *MFF*, respectively) and mitophagy (*PRKN2*). We considered to analyse the same cells used for the mitochondrial DNA copy number evaluation, in order to perform an adequate correlation. This study was performed in a subgroup of 30 SLE patients and 15 healthy controls, randomly selected from the initial cohort.

**Fig. 3:**
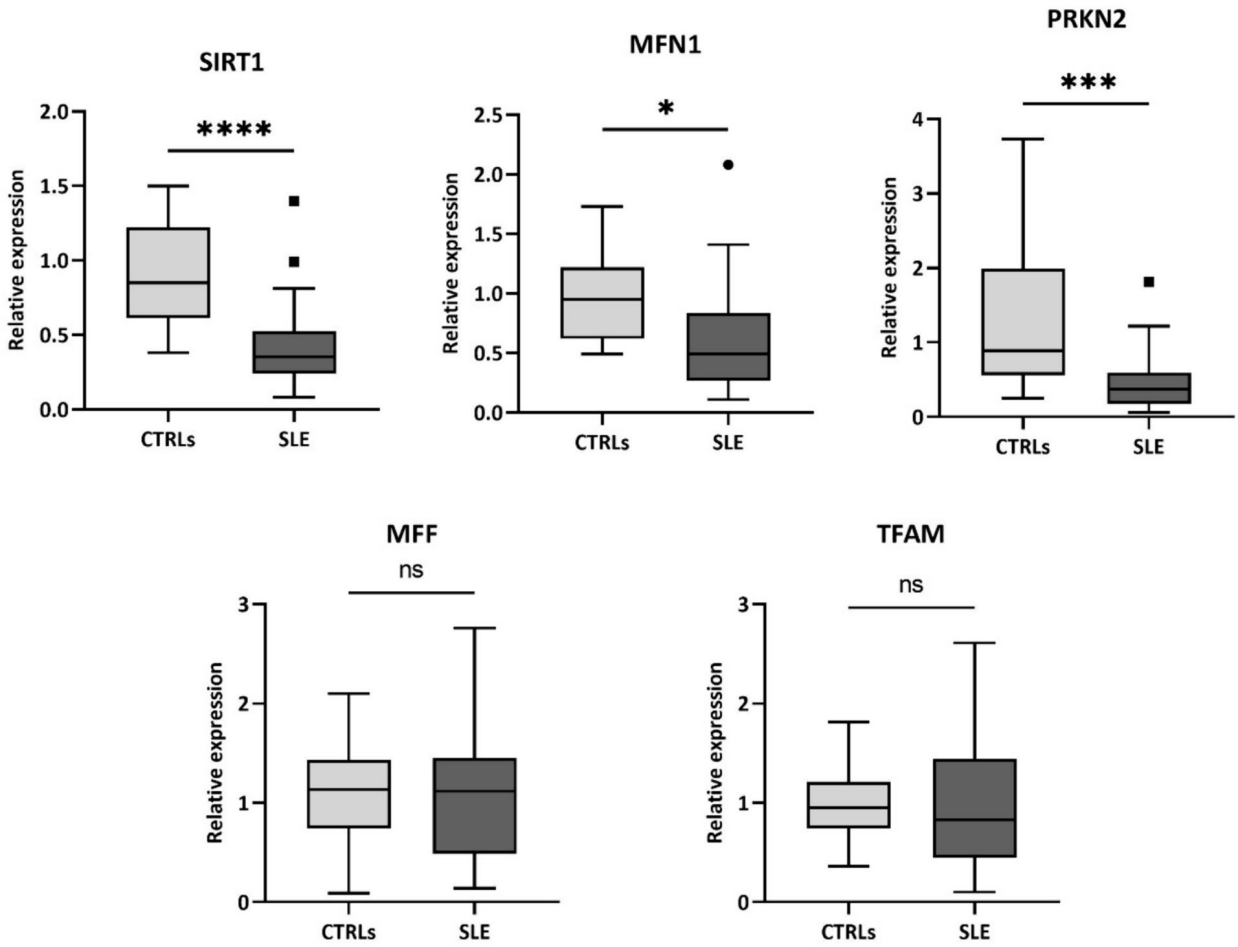
Comparisons of gene expression levels between healthy controls (CTRLs) and patients with Systemic Lupus Erythematosus (SLE). ****P < 0.0001; *P = 0.015; ***P = 0.0009; ns, not significant

Expression levels of *SIRT1*, *MFN1* and *PRKN2* genes in SLE patients and healthy controls are shown in Fig. 3.

Our results showed a significant decrease in mRNA expression levels of *SIRT1*, *MFN1* and *PRKN2* in SLE patients when compared to the controls (*P* = 0.00001 for SIRT1, *P* = 0.0150 for *MFN1* and *P* = 0.0009 for *PRKN2*). Instead, no significant differences in the expression levels of *TFAM* and *MFF* were found.

Subsequently, we have explored possible associations between the expression levels of the genes, which emerged as altered from the previous analysis, and the clinical/laboratory data (Table 2).

**Table 2:** Associations between the expression levels of *MFN1* and *PRKN2* genes and the clinical/laboratory data of SLE patients

Gene	Clinical/laboratory data	Evidence
*MFN1*	Leukopaenia	Up-regulation
	RNP antibodies	
	Anti-Sm antibodies	
	Lower C3 antigen concentrations	
*PRKN2*	Thrombocytopaenia	Up-regulation

**Fig. 4:**
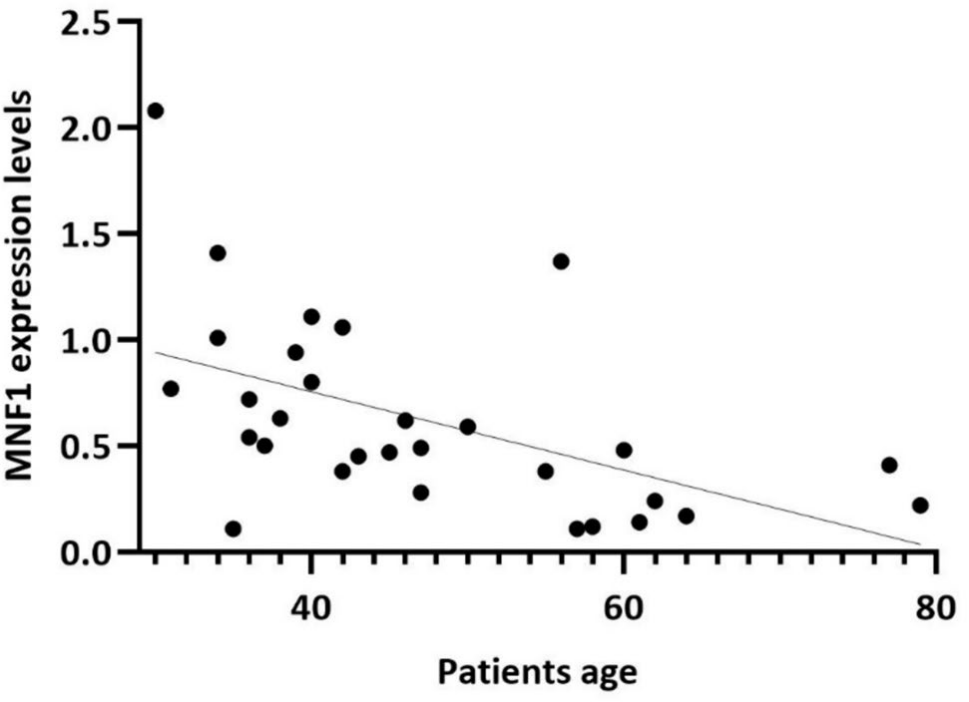
Correlation analyses between *MNF1* expression levels and patients’ age

Patients with leukopaenia (*P* = 0.016), RNP antibodies (*P* = 0.001) and anti-Sm antibodies (*P* = 0.015) detectable and lower C3 antigen concentrations (*P* = 0.03) had higher expression levels of *MFN1*. In addition, a negative correlation between the expression of *MNF1* and patients’ age was observed (*P* = 0.003, *r* =  − 0.527) (Fig. 4).

Moreover, a significant association was found between high *PRKN2* mRNA expression levels and thrombocytopaenia (*P* = 0.035).

### Correlation analysis

**Fig. 5:**
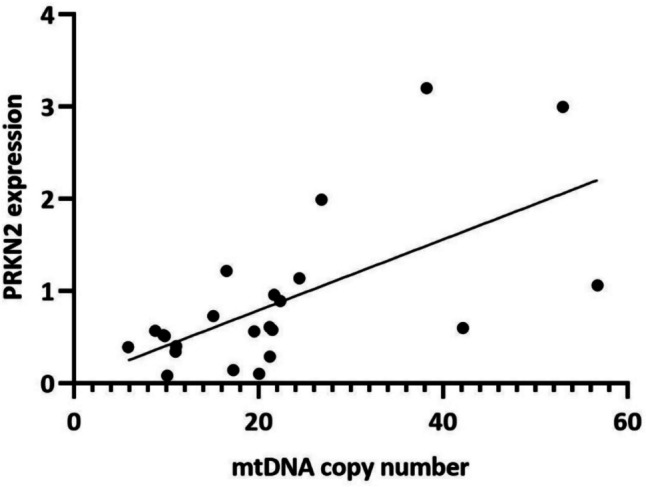
Correlation analyses between *PRKN2* expression levels and mtDNA copy number

We analysed the linear correlation among the expression of mitochondrial homeostasis genes and we observed that *MFN1* transcript levels positively correlate with *MFF* (*P* = 0.003, *r* = 0.431) and *TFAM* (*P* = 0.001, *r* = 0.469). In addition, we also observed a positive correlation between the mRNA levels of *SIRT1* and *PRKN2* (*P* < 0.001; *r* = 0.602), accordingly with the observed decrease of the two genes in SLE patients. Moreover, both *SIRT1* and *PRKN2* mRNA expression positively correlated with the markers of mitochondrial *MFF* fission gene (*P* = 0.049, *r* = 0.299 for *SIRT1*; *P* = 0.041, *r* = 0.317 for *PRKN2*) and *MFN1* fusion gene (*P* = 0.002, *r* = 0.463 for *SIRT1*; *P* = 0.010, *r* = 0.396 for *PRKN2*). Lastly, we evaluated if the expression levels of analysed genes correlated with mtDNA copy number and we found a positive correlation with *PRKN2* expression level (*P* = 0.019, *r* = 0.475) (Fig. 5).

## Discussion

Mitochondria are important organelles that play a key role in many cellular processes; in particular, they are involved in energy production (ATP) through oxidative phosphorylation. Structural damage and functional alterations of mitochondria cause various pathological states, including oxidative stress and inflammatory reactions. Mitochondrial dysfunction and increased OS have been identified as a critical pathogenetic factor in T cell-mediated autoimmune disorders, such as SLE [25]. Since mtDNA copy numbers could change in response to physiological or environmental stimuli, like the increased OS, we have evaluated the mtDNA copies in PBMCs of a cohort of SLE patients. In this study, we have observed that SLE patients exhibited a significantly lower amount of mtDNA copy number compared to the healthy controls. These data are in accordance with the evidence in the literature that had described a decrease in mtDNA content in diseases associated with OS, including SLE [14, 15]. Furthermore, we described a higher mtDNA content in SLE patients positive for LAC antibodies; this result is not surprising because it is known that antiphospholipid antibodies affect mitochondria dynamics, favouring the fission rate [26]. In addition, we described a higher mtDNA content in SLE patients positive for anti-RNP antibodies; it is known that, when mtDNA is damaged, it undergoes degradation by lysosomes and this step requires the dissociation of TFAM from the mtDNA. A recent study on SLE patients has highlighted that an exposure of neutrophils to anti-RNP antibodies interferes with the degradation process in the lysosome, resulting in mtDNA retention [27]. We could suppose that a similar mechanism also occurs in our SLE cohort, explaining the higher mean value of mtDNA copy number in patients positive for anti-RNP antibodies with respect to negative ones. Further studies are required to confirm these hypotheses. In fact, understanding the role of SLE antibodies in inducing mitochondrial changes could be useful to use them as biomarkers to stratify SLE patients.

Then, we have investigated the expression variability of the most common genes involved in mitochondrial homeostasis to verify if the alteration of mitochondria number and morphology could be linked to their dysregulation. We found that SLE patients have statistically lower mRNA levels for *SIRT1*, *MFN1* and *PRKN2* genes, compared with the controls. *SIRT1* gene encodes for a protein belonging to the sirtuin family, a class III histone deacetylase [28]. SIRT1 is known to be involved in mitochondrial biogenesis through deacetylation of PGC-1α (peroxisome proliferator-activated receptor-gamma coactivator); deacetylate PGC-1α enters in the nucleus which promotes mitochondrial biogenesis and increases the number and activity of mitochondria [16, 29]. Recent studies have also reported that SIRT1 suppresses mitochondrial OS, reducing ROS production [30]. We could suppose that decreased expression of *SIRT1* affects the biogenesis process in SLE patients, blocking the mitochondrial turnover, and this leads to fewer vital mitochondria and increased ROS production. Our results showed also a positive correlation of mRNA levels of *SIRT1* with fission and fusion mitochondrial markers, and this seems to confirm the hypothesis of a dysregulation of mitochondrial dynamics.

*MFN1* gene encodes for a transmembrane GTPase responsible for the fusion process that is involved in the control of the number of healthy mitochondria and, consequently, in the decrease of the ROS production [18]. It is reported that mouse embryonic fibroblast knockout for Mfn1 has highly fragmented mitochondria with respect to the physiological tubular network observed in wild-type cells [31, 32]. Since mitochondrial morphology is regulated by a balance between fusion and fission pathways, the decreased expression of *MFN1* gene could affect this equilibrium, favouring a more fragmented morphology of mitochondria in SLE patients. In addition, fragmented mitochondria induced by the knockdown of fusion proteins also increased mitochondrial ROS production [33]. Moreover, we have observed a negative correlation between the expression of *MNF1* and patient’s age; some evidence tied the alteration of mitochondrial dynamics to specific cellular and molecular hallmarks of aging [34], so it is not surprising that the dysregulation of the fusion process increases with age.

Lastly, *PRKN2* gene encodes for an E3 ubiquitin ligase (Parkin) that is involved in various aspects of mitochondrial functioning, including the mitophagy process [19]. This process is a particular type of autophagy that eliminates dysfunctional mitochondria. Impaired mitophagy leads to the accumulation of dysfunctional mitochondria and disruption of their function; this is associated with several diseases, such as neurodegenerative and cardiovascular diseases, and could also affect innate immunity [35]. The defective removal of damaged mitochondria, due to decreased expression of *PRKN2* gene, contributes to the activation of inflammatory signalling pathways and the development of chronic systemic inflammation. Moreover, we also reported a positive correlation between the mRNA levels of *SIRT1* and *PRKN2* in PBMCs of SLE patients; it is known that Parkin expression is regulated via SIRT1 and, together, promotes the mitophagy process [36, 37]. In addition, our results have described a positive correlation between mtDNA copy number and *PRKN2* expression levels. A decrease in *PRKN2* expression could contribute to an increased number of damaged mitochondria, which generates other ROS that affect the number of functional mitochondria; in fact, it is reported that parkin-deleted fibroblasts of Parkinson patients showed a reduction of the mtDNA copy number [38]. So, we could hypothesize that the decreased amount of mtDNA copy number, observed in SLE patients, is due to an accumulation of ROS generated by damaged mitochondria.

Despite these results, our study has some limitations; the first is the lack of data regarding the measurement of ROS production in our SLE patients, related to insufficient available material. A second limitation is that all the analyses were performed on PBMCs and not in a specific cellular sub-population. Therefore, it will be interesting to perform further analysis on oxidative stress markers and replicate the study on sub-populations of PBMCs.

**Fig. 6:**
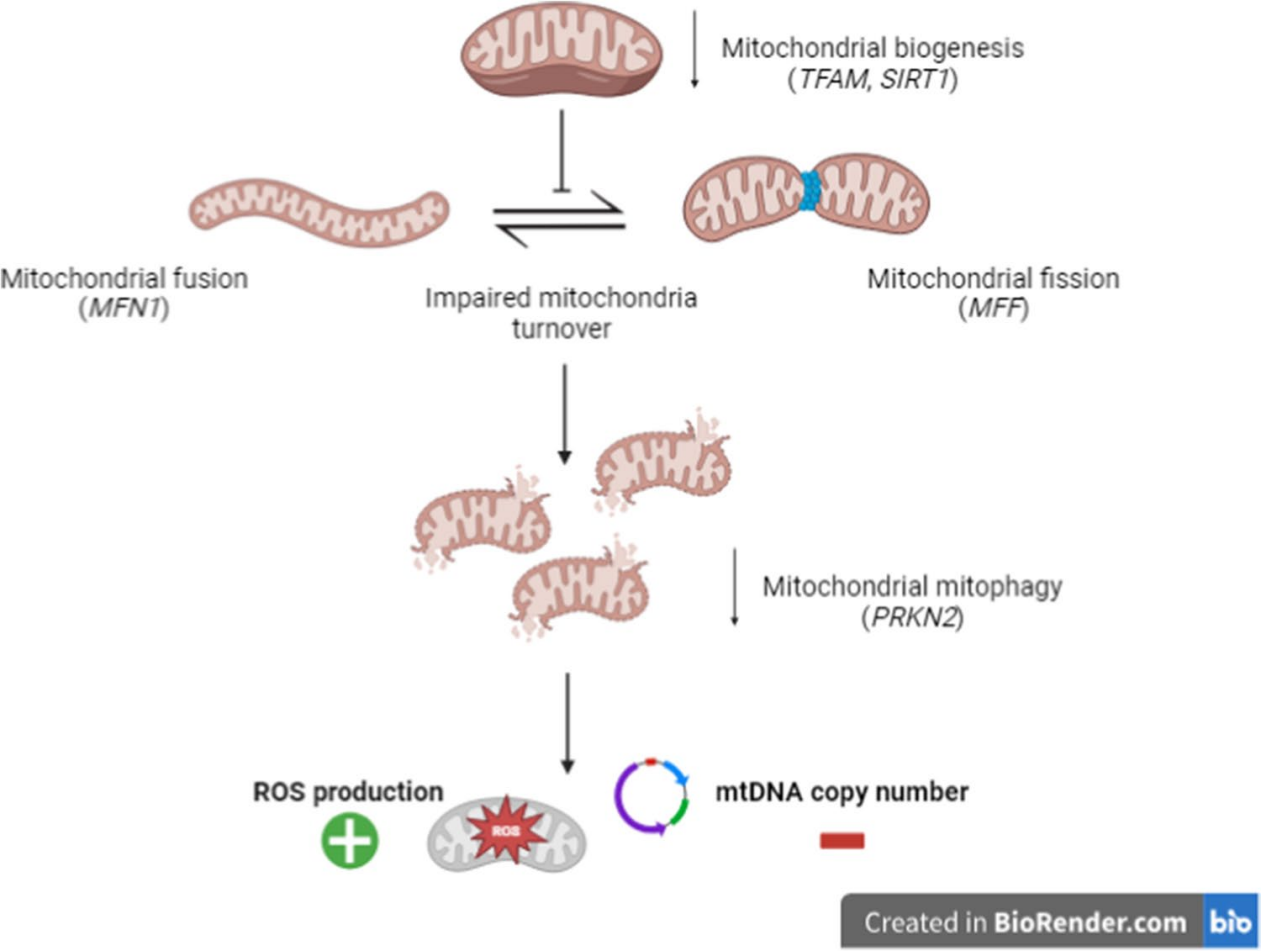
Dysregulation of mitochondrial homeostasis in SLE patients (in brackets, studied genes involved in different steps)

In Fig. 6, we have summarized our results and shown the possible mechanism of dysregulation of mitochondrial homeostasis in SLE patients (Fig. 6).

## Conclusion

In conclusion, our data confirm a quantitative alteration of mtDNA in SLE patients and describe a significant decrease in expression levels of genes involved in mitochondrial homeostasis. If confirmed and supported by functional studies, these results could provide additional support to the important role played by mitochondria in the development of SLE. Targeting mitochondrial dysfunction and oxidative stress may offer promising therapeutic avenues for treating SLE and other autoimmune diseases.

## Data Availability

No datasets were generated or analysed during the current study.
